# The effects of acupuncture on occipital neuralgia: a systematic review and meta-analysis

**DOI:** 10.1186/s12906-020-02955-y

**Published:** 2020-06-03

**Authors:** Jung-Min Yun, Sook-Hyun Lee, Jae-Heung Cho, Koh-Woon Kim, In-Hyuk Ha

**Affiliations:** 1 College of Korean Medicine, Woosuk University, Wanju, Republic of Korea; 2 Jaseng Spine and Joint Research Institute, Jaseng Medical Foundation, Seoul, Republic of Korea; 3 Department of Korean Rehabilitation Medicine, Kyung Hee University, Seoul, Republic of Korea

**Keywords:** Occipital neuralgia, Acupuncture, Systematic review, Meta-analysis, The level of evidence

## Abstract

**Background:**

Occipital neuralgia is one of the main causes of occipital pain. This systematic review aims to assess the level of evidence in randomized controlled trials (RCTs) on the effects of acupuncture on occipital neuralgia.

**Methods:**

We searched 11 databases and a journal archive from their inception up to December 2019 for relevant RCTs. We did not place any specific restrictions on patients diagnosed with occipital neuralgia, such as age or gender. We included studies that used an acupuncture intervention group, with or without the control group treatment, and that set a control group receiving active, interventional treatment such as medication. For outcomes, we used visual analogue scale (VAS) and effective rate.

**Results:**

We included a total of 11 RCTs. All VAS scores (mean difference [MD] –2.35, 95% confidence interval [CI] –2.84, − 1.86) and effective rate values (odds ratio [OR] 4.96, 95% CI 2.24, 10.96) showed significant differences in effect between acupuncture treatment alone and the control group treatment. Similarly, combined acupuncture treatment with control group treatment also showed significant effects in effective rate (OR 6.68, 95% CI 1.11, 40.37). We performed a subgroup analysis on studies that used acupuncture only as the intervention and reported the effective rate, and found that all acupuncture subgroups showed significant effects compared to the control group treatments. None of the studies reported severe adverse effects.

**Conclusions:**

Although acupuncture only and combined acupuncture treatments showed significant effects compared to medication, the results of this study are inconclusive. Studies with rigorous study design and larger sample sizes are needed to confirm the role of acupuncture in this field.

**Trial registration:**

International prospective register for systematic review (PROSPERO) number CRD42019128050.

## Background

Occipital neuralgia is a disease characterized by unilateral or bilateral sudden, sharp, stabbing pain. It is a headache disease sometimes accompanied by sensory abnormalities or loss of sensation, and associated with abnormalities in the greater, lesser or third occipital nerves in the occipital area [1]. The greater and lesser occipital nerves usually arise from the dorsal ramus of cervical spinal nerve 2. The greater occipital nerve passes the inferior margin of obliquus capitis inferior, which forms the inferior border of the suboccipital triangle, ascends through semispinalis capitis, and emerges to the scalp at the attachment of the trapezius to the occipital bone [2]. Meanwhile, the lesser occipital nerve ascends along the posterior margin of sternocleidomastoid and then branches as it approaches the scalp, innervating the area lateral to the distribution of the greater occipital nerve [3]. The third occipital nerve arise from cervical spine nerve 3 and innervates occipital region [3]. Pressure, inflammation, and friction along the trajectories of these nerves are known to contribute to the development of occipital neuralgia.

According to Koopman et al. (2009) [4], around 3.2 out of 100,000 people experience occipital neuralgia. Thus, since the incidence of occipital neuralgia is not especially high, it is difficult to establish diagnostic criteria [5]. In fact, the ICHD-3 provides diagnostic criteria for occipital neuralgia, but these are of limited usefulness, since the criteria consist simply of subjective pain or sensory abnormalities in areas related to nerves in the occipital region, and there is still a lack of objective criteria.

The latest treatment for occipital neuralgia often uses methods based only on clinical experience or case studies, because there is not yet any clear consensus on treatments for occipital neuralgia. This is believed to be related to the lack of randomized controlled trials (RCTs) for most treatment modalities [6]. Typically, occipital neuralgia is treated conservatively, using anti-inflammatory analgesics or anti-depressants, but if there is no response to conservative treatment, occipital nerve block may also be used [7]. In addition, occipital neuralgia is often accompanied by other headaches that respond poorly to treatment, such as chronic headache or migraine, and so occipital nerve block is performed after trying several other headache treatment methods [8].

Of the various TOM treatments which include acupuncture, pharmacoacupuncture, acupotomy, and chuna (tuina) therapy, acupuncture has been reported to be better than drugs at treating occipital neuralgia in terms of pain relief and effective rate [9–14]. Specifically, acupuncture treatment at the points BL10, GV13, BL11, LU6, and SI3 is effective and ear acupuncture, pharmacopuncture, fire needle acupuncture, and electroacupuncture may be used [15]. Since acupuncture can be considered as an alternative treatment method to avoid the adverse effects of typical medication using anti-inflammatory analgesics and anti-depressants, we believe that acupuncture can be an important treatment for occipital neuralgia.

Studies on the effects of acupuncture on occipital neuralgia have mostly consisted of case studies and RCTs. A large number of occipital neuralgia patients have been treated using acupuncture at TOM institutions in Korea, but there are relatively few papers providing evidence for the effects of acupuncture, and there has still been no systematic review either domestically or overseas. Hence, in this paper, we aimed to conduct a systematic review of previous clinical trials using acupuncture to treat occipital neuralgia, in order to explore and present evidence for the effects of acupuncture.

## Methods

The protocol of the current study was registered in the PROSPERO International prospective register of systematic reviews (CRD42019128050). This systematic review was conducted and is reported according to the Preferred Reporting Items for Systematic Reviews and Meta-Analyses (PRISMA) guidelines (2009) [16].

### Criteria for inclusion and exclusion

The criteria for inclusion and exclusion were as follows: (1) study types: a RCT that was reported in English, Chinese, and Korean; (2) participants: we selected patients diagnosed with occipital neuralgia regardless of their age, sex, or the duration of illness and described as such (terms in parenthesis). However, we excluded studies on patients with the occipital headache irrelevant with the lesions of occipital nerves; (3) interventions: in terms of the acupuncture treatment used as the intervention, there were no restrictions on the sub-classifications based on the technique or location of acupuncture. Additionally, studies in which the control treatment was used alongside acupuncture in the intervention group were included. However, we excluded studies that used dry needling, pharmacopuncture, and electroacupuncture in the intervention group; (4) controls: in terms of the control groups, we not only included studies with non-treatment groups, placebo groups, or clinical treatment groups, but also those using active interventional treatments, such as medication. However, when the control group only underwent a single treatment modality, we excluded studies where that treatment was acupuncture, such as those only comparing different types of acupuncture. We also excluded studies in which the control group underwent combination therapy, making it impossible to investigate the effects of acupuncture alone; (5) outcomes: we used the visual analogue scale (VAS) and some estimating measures, such as effective rate, which could be calculated objectively. The VAS is the most commonly used scale to efficiently measure headache; the extent of pain is indicated as a number between the fixed points of 0, indicating “no pain”, and 10, indicating “the most severe pain imaginable” [17]. The effective rate was calculated as the ratio of patients showing complete or partial improvement to the patients showing no improvement. However, we excluded articles that did not report outcome indices relevant to this study. Adverse events were also selected for our search.

### Search strategy

We searched PubMed, EMBASE, the Cochrane Central Register of Controlled Trials, Allied and Complementary Medicine Database (AMED), AcuTrials, the China National Knowledge Infrastructure (CNKI), and Japan Science and Technology Information Aggregator Electronic (J-stage) from database inception to December 2019. For Korean publications, we searched 4 Korean medical databases (the Research Information Service System, National Discovery for Science Leaders, Korean Studies Information Service System, and Oriental Medicine Advanced Searching Integrated System). We also searched for conference papers in the Journal of Acupuncture Research. The search was performed without restrictions in the year of publication.

We selected search terms to search for studies related to acupuncture treatment for occipital neuralgia. The search terms included were “occipital neuralgia”, “acupuncture” AND “random” in each database language, and the references cited by the searched articles were also tracked. (Additional file 1). Moreover, we did not restrict the details of the intervention, such as the type of needle inserted, angle at which it was inserted, depth, number, duration, and acupoints.

### Article screening

We used base search terms referring to the primary intervention, acupuncture, and the disease of interest, i.e., occipital neuralgia; then, among the retrieved studies, two authors (JM and SH) independently selected studies to include in the analysis based on a review of the title, abstract, and main text. After the authors independently confirmed whether studies met the criteria for inclusion, they cross-verified their work. Any disagreements over the selected studies were resolved by discussing the issues with a third author and reaching a consensus.

### Data extraction

Shared data were extracted from each study based on a consensus between two authors (JM and SH) after each search was conducted independently; authors, year of publication, sample size, intervention, major outcomes, and adverse effects were considered shared data. Any disagreements between the authors over the eligibility of particular data were resolved by discussing the issues with a third author and reaching a consensus.

### Data collection and analysis

This was a systematic literature review of studies examining the effects of acupuncture on occipital neuralgia. From the studies retrieved by searching, two authors (JM and SH) first selected and excluded studies based on the title and abstract, and then reviewed the main text of the selected studies to choose the final studies to include in the analysis. From the studies finally selected by the two authors, information such as the authors, year of publication, sample size, intervention, and major outcomes, and this information was summarized in a table.

The effects of acupuncture in the selected studies were analyzed using Cochrane Collaboration software [Review Manager (RevMan) Version 5.3 for Windows. Copenhagen: The Nordic Cochrane Centre]. The outcome variable, VAS, was continuous data, and was expressed in terms of the mean difference (MD) and 95% confidence interval (CI) using inverse variance estimation. The other outcome variable, effective rate, was dichotomous data, and was expressed in terms of the odds ratio (OR) and 95% CI using the Mantel-Haenszel estimation method. A random effects model was applied to all analyses. The heterogeneity of the studies was analyzed using a chi-square test (test with *p*-value of *p* < 0.10) and Higgins I^2^ statistic. In the case that statistically significant heterogeneity was observed, subgroup analysis was performed to analyze the cause of heterogeneity. In the case that there were 10 or fewer studies in each group, we did not assess publication bias.

### Methodological quality assessment for the included studies

#### Risk of bias

Two independent researchers assessed the risk of bias in the selected studies based on the seven items presented in the Cochrane Handbook’s method of assessing risk of bias [18]. The risk of bias was assessed independently by two reviewers (JM and SH). The risk of bias of each examined study was assessed in six areas of trial design (sequence generation, allocation sequence concealment, blinding of participants and personnel, blinding of outcome assessment, incomplete outcome data, selective outcome reporting) with ranking each area as high, low or unclear. If any disagreements happened in evaluating process, they were resolved by discussion.

#### The level of evidence

The level of evidence regarding study outcomes was analyzed with reference to GRADE (The Grading of Recommendations, Assessment, Development and Evaluation) [19]. Studies were assessed in the following categories: risk of bias, imprecision, inconsistency, indirectness, publication bias, large magnitude of effect, dose-response, and confounding. The results of the assessment were assigned one of four grades: high, moderate, low, or very low.

## Results

### Description of studies

**Fig. 1:**
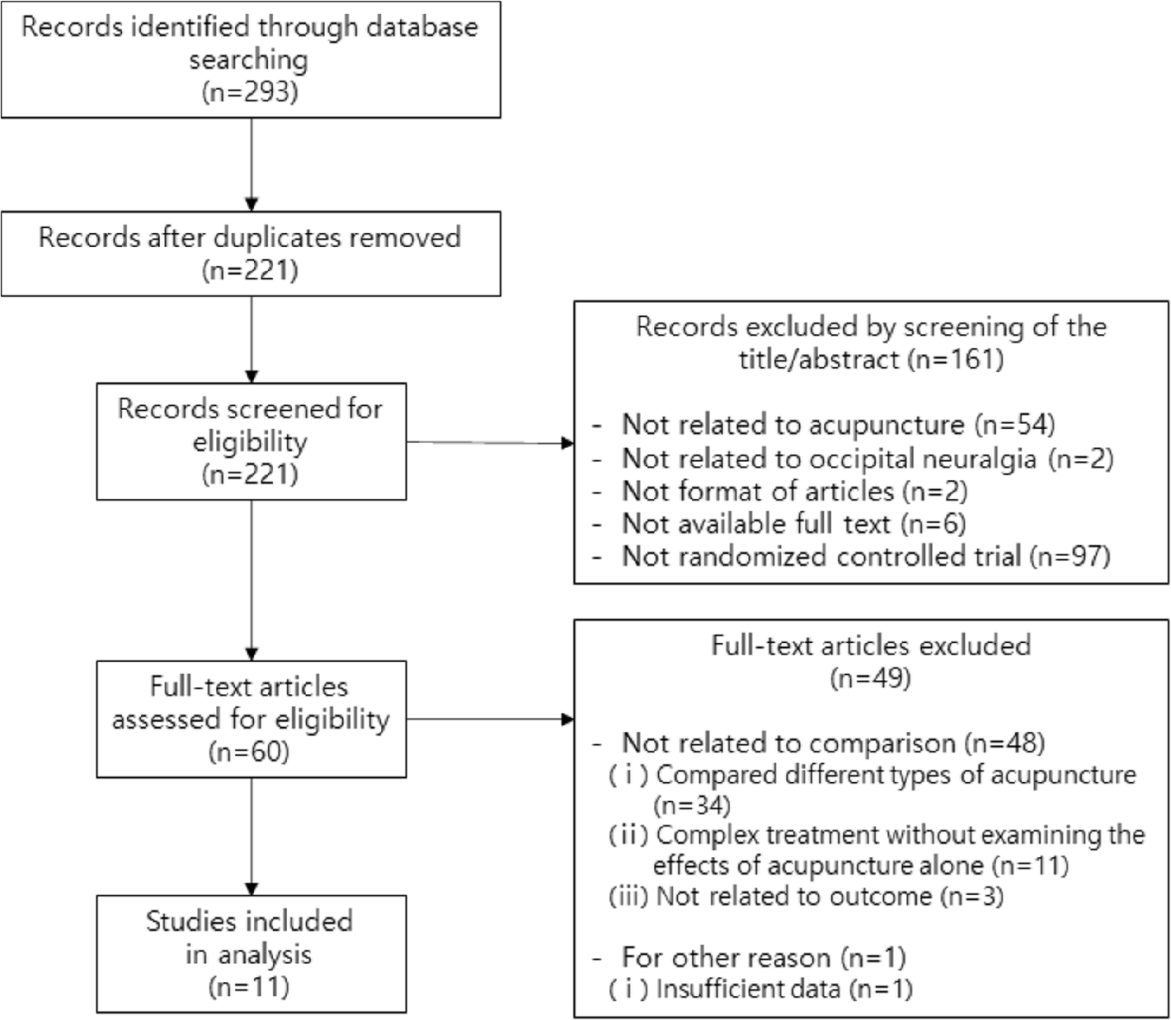
Flow chart of the trial selection process

The total of 293 studies were retrieved from searching 11 databases and 1 journal. We first excluded 72 duplicate studies; after reviewing the titles and abstracts, a further 161 studies were excluded because they were not related to acupuncture, because the patients did not have occipital neuralgia, because they were not in the form of academic papers, because we were unable to access the main text, or because they were not RCTs. After a detailed review of the main text, 49 studies were excluded because the control group was not appropriate, or because a lack of data made it impossible to determine the precise results. Finally, 11 studies were selected for our analysis (Fig. 1).

We performed quantitative and qualitative analysis of the 11 studies. Of these, 6 studies used VAS as an endpoint and 10 studies used effective rate as an endpoint. Thus, we performed a quantitative meta-analysis on these studies.

The 11 finally selected RCTs all used acupuncture only or acupuncture combined with the control group treatment as their intervention, while the control groups were treated with medication or using a different acupuncture method to the intervention group [13, 14, 20–28]. There were 9 studies using only acupuncture as the intervention, and 2 studies using acupuncture in combination with the control group treatment. Specifically, the most common form of acupuncture was reinforcing and reducing manipulation (補瀉), followed by the manipulation causing a special sensation called “*de qi* (得氣),” while there were also studies that used the twirling method (捻轉), the red-hot needle method (火針), or the lifting method (提揷). The most commonly used acupuncture points were GB 20 (風池) and the *ashi* points (阿是穴), followed by EX-B2 (夾脊穴). The detailed information for each study has been summarized in a table (Table 1).

**Table 1:** Summary of Randomized Controlled Trials of acupuncture for Occipital neuralgia

Author (year)	Condition Sample size	Gender (M/F)	Age	Intervention Group	Control Group	Main Outcomes
***Acupuncture*vs*Control (Medication)***						
Chen2014	Occipital neuralgia (37/37)	I: 20/17;	I: 44.4 ± 11.0;	A) AT (Ashi points; 1–2 times a day for 3 days, 20–30 min; Reducing manipulation)	B) Medication treatment	1) VAS
		C: 19/18	C: 46.2 ± 10.7			2) Effective rate
Li2016	Occipital neuralgia (29/29/25)	I: 12/17;	I: 36 ± 9.3;	A) AT (GB20, EX-B2, Anmyeon points; 1 time a day for 6 days, 30 min; Proximal-needle, Reinforcing-Reducing manipulation, Technique for getting *de qi*)	B) AT (Conventional-needle, Reinforcing-Reducing manipulation, Technique for getting *de qi)*	1) VAS
		C1: 14/15;	C1: 39 ± 8.4;			2) Effective rate
		C2: 11/14	C2: 40 ± 9.7			3) Recurrence rate
					C) Medication treatment	
Ning2012	Occipital neuralgia (30/30)	I: 12/18;	I: 53.2 ± 12.358;	A) AT (BL9, GB20, GB12, EX-B2, EX-HN14, Ashi points; 1 time a day for 6 days, 20 min; Reinforcing-Reducing manipulation)	B) Medication treatment	1) VAS
		C: 11/19	C: 51.6 ± 12.478			
Cui2011	Occipital neuralgia (30/30/30)	I: 13/17;	I: 28–65;	A) AT (GB20, EX-HN14, EX-B2, Ashi points; 1 time a day for 20 days, 22 min; Twirling manipulation)	B) AT (『针灸治疗学』 method, Twisting method, Technique for *getting de qi*)	1) VAS
		C1: 16/14;	C1: 25–67;			2) BRS-6
		C2: 12/18	C2: 26–66			3) Effective rate
					C) Medication treatment	
Hong2014	Occipital neuralgia (30/27)	I: 14/16;	I: 45.1 ± 13.4;	A) AT (Ashi points; 1 time a day for 5 days; Fire needle, Reinforcing-Reducing manipulation)	B) Medication treatment	1) VAS
		C: 12/15	C: 46.0 ± 12.9			2) Effective rate
Gao2016	Occipital neuralgia (30/30)	I: 10/20;	I: 51.8;	A) AT (GB8, GV20, GB20, LI4, PC6, LU7, LR3, EX-HN1, EX-HN3, EX-HN5, EX-B2; 1 time a day for 28 days, 30 min; Twirling manipulation, Lifting manipulation, Reinforcing-Reducing manipulation)	B) Medication treatment	1) Effective rate
		C: 8/22	C: 50.9			
Liu2006	The Greater Occipital neuralgia (30/30)	I: 19/11;	I: 25–57;	A) AT (GB18, GB39, Ashi points; 1 time a day for 10 days, 30 min; Reinforcing-Reducing manipulation, Technique for getting *de qi*)	B) Medication treatment	1) Effective rate
		C: 17/13	C: 22–53			
Yang2016	Occipital neuralgia (38/37)	I: 22/16;	I: 54.32 ± 10.53;	A) AT (GB20, TE17, GB12, GV14, LI4, TE5, LR3, KI3, ST40, SP10, ST36, SP6, EX-B2, EX-HN5, Ashi points; 1 time a day for a week, 30 min; Fire needle, Reinforcing-Reducing manipulation)	B) Medication treatment	1) Effective rate
		C: 23/14	C: 58.77 ± 11.61			2) Daily pain time index
Lin2005	Occipital neuralgia (35/32)	I: 21/14;	I: 46;	A) AT (Wrist-Ankle region; 1 time a day for 10 days; Needle-embedding therapy)	B) Medication treatment	1) Effective rate
		C: 19/13	C: 47			
***Acupuncture + Control (Medication)*vs*Control (Medication)***						
Xu2014	Occipital neuralgia (32/30/30)	I: 14/18;	I: 49.25;	A) AT + Medication treatment (GV20, GB20, EX-B2, Ashi points; 1 time a day for 20 days, 22 min)	B) AT (Same with intervention)	1) VAS
		C1: 13/17;	C1: 50.23:			2) Effective rate
		C2: 12/18	C2: 48.94		C) Medication treatment	
Li2007	Occipital neuralgia (34/34)	I: 16/18;	I: 37.7;	A) AT + Medication treatment (GB20, BL10, GB8, ST8, GB5, EX-HN5; 1 time a day for 6 days, 30 min; Reinforcing-Reducing manipulation, Technique for getting *de qi*)	B) Medication treatment	1) Effective rate
		C: 15/19	C: 38.6			

Abbreviations: AT: Acupuncture Treatment, VAS: Visual Analogue Scales, BRS-6: the 6-point Behavioral Rating Scale, adverse effects were not reported for any study

### Risk of bias assessment

**Fig. 2:**
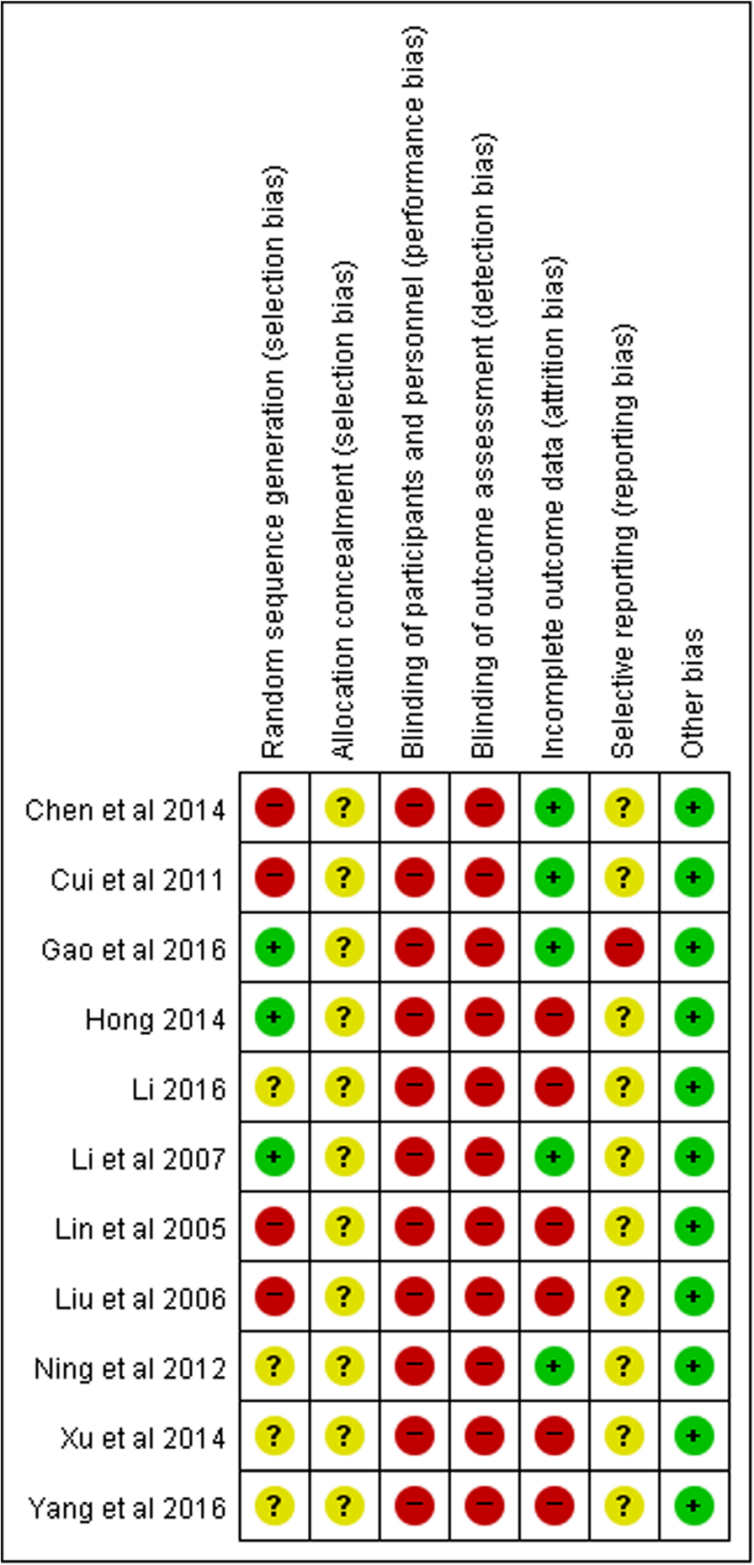
Risk of bias assessed using the Cochrane “Risk of bias” tool. +, low risk of bias; −, high risk of bias;?, unclear risk of bias

We assessed the risk of bias in the 11 RCTs by applying the Cochrane Risk of Bias criteria in Fig. 2 [18].

#### Random sequence generation

There were 4 studies that allocated subjects to the treatment or control group based on the order of diagnosis and treatment, showing a high risk of bias [13, 21, 24, 26]. There were 3 studies that showed a low risk of bias because they used a random number table or drawing of lots [22, 23, 28]. The other 4 studies were judged to show unclear risk, since they did not describe the group allocation method.

#### Allocation concealment

All 11 studies were judged to show unclear risk, since they did not describe the method of allocation concealment.

#### Blinding of participants and personnel

All 11 studies were judged to have a high risk of bias with regard to blinding due to the nature of RCTs on acupuncture.

#### Blinding of outcome assessment

All 11 studies were judged to have a high risk of bias with regard to blinding of outcome assessments.

#### Incomplete outcome data

There were 6 studies judged to show a high risk of bias because patient group selection/exclusion seemed to have affected the outcomes, or because there was no comparison of demographic characteristics between the treatment group and the control group [20, 22, 24, 26–28]. The other 5 studies were judged to show low risk of bias.

#### Selective reporting

In the study by Gao et al., 2016, although the effective rate was assessed using VAS based on the researchers’ own criteria, specific VAS results were not given, and so the risk of bias was judged to be high [23]. All other studies were judged to show unclear risk, since there were no reports regarding adverse effects of acupuncture used as the intervention or of drug treatment.

#### Other bias

All studies were judged to show low risk of other bias.

### Effects of interventions

**Fig. 3:**
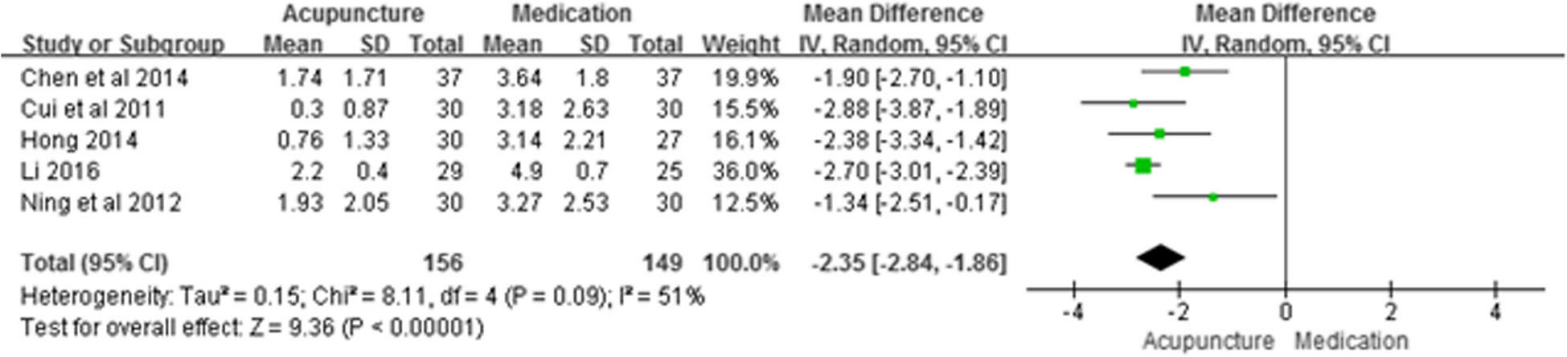
Meta-analysis of Acupuncture vs. Medication (VAS)

There were a total of 5 studies that used acupuncture as the intervention, medication as the control group, and presented VAS results; we used these studies in a meta-analysis of the endpoint VAS (Fig. 3). The results showed an MD of − 2.35 (95% CI –2.84, − 1.86; *P* < 0.001; *n* = 305; I^2^ = 51%), indicating that acupuncture was more effective at treating occipital neuralgia than medication.

**Fig. 4:**
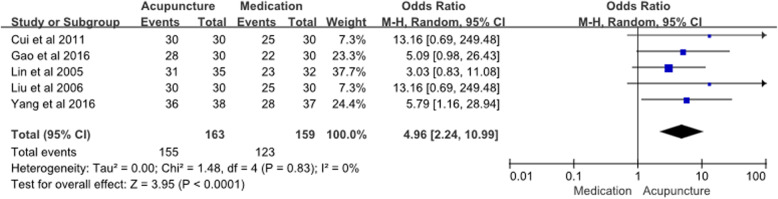
Meta-analysis of Acupuncture vs. Medication (Effective rate)

There were 5 studies using acupuncture as the intervention, medication as the control group, and presenting effective rate results; we used these studies in a meta-analysis of the endpoint effective rate (Fig. 4). The OR for acupuncture compared to medication for occipital neuralgia was 4.96 (95% CI 2.24, 10.96; *P* < 0.001; *n* = 322; I^2^ = 0%), indicating that acupuncture is more effective. There were 2 studies using acupuncture combined with medication as the intervention, medication only as the control group, and presenting effective rate results; we used these 2 studies to perform a meta-analysis (Additional file 2). The OR for acupuncture compared to medication for occipital neuralgia was 6.68 (95% CI 1.11, 40.37; *P* = 0.04; *n* = 130; I^2^ = 0%), indicating that combined acupuncture with medication is more effective than medication alone. There were 3 studies that used acupuncture as the intervention and medication as the control; however, we could not calculate the effective rate from their data.

For a more precise analysis, we collected some information of acupuncture treatment in detail. (Table 1). We selected the studies that compared acupuncture only with medication and presented the effective rate, and divided these into subgroups based on 3 criteria: the number of acupuncture points, the type of acupuncture points, and the total treatment duration. We then performed a subgroup analysis. When we analyzed the subgroups with < 10 or ≥ 10 acupuncture points, acupuncture was significantly more effective than medication in both subgroups, with ORs of 4.57 (95% CI 1.52, 13.72; *P* = 0.007; *n* = 187; I^2^ = 0%) and 5.44 (95% CI 1.72, 17.19; *P* = 0.004; *n* = 135; I^2^ = 0%) for < 10 and ≥ 10 acupuncture points, respectively (Additional file 3). In the analysis by type of acupuncture points, acupuncture based on meridian theory was more effective than medication with an OR of 6.69 (95% CI 2.44, 18.31; *P* < 0.001; *n* = 255; I^2^ = 0%), and acupuncture not related to meridian theory was more effective than medication with an OR of 3.03 (95% CI 0.83, 11.08; *P* = 0.09; *n* = 67; Additional file 4). In the analysis by treatment duration, acupuncture treatment for period ≤1 week showed an OR of 5.79 (95% CI 1.16, 28.94; *P* = 0.03; *n* = 75), acupuncture for 8–14 days showed an OR of 3.85 (95% CI 1.18, 12.59; *P* = 0.03; *n* = 127; I^2^ = 0%), and acupuncture for > 2 weeks showed an OR of 6.39 (95% CI 1.52, 26.87; *P* = 0.01; *n* = 120; I^2^ = 0%), all of which were significantly more effective than medication (Additional file 5).

### Adverse events reporting

None of the selected studies reported any particular adverse events.

### The level of evidence

Table 2 shows the level of evidence for each meta-analysis. The studies used in the analyses had a small number of subjects, and were not properly blinded, which increased the imprecision and the risk of bias in all analyses. In the comparisons of VAS and effective rate between acupuncture and medication, group allocation was not properly designed, which incurred further penalties in terms of the risk of bias. While the analysis of VAS showed a very low level of evidence, the analysis of effective rate showed a low level of evidence due to the larger effect size in the intervention groups. Most subgroup analyses comparing the effective rate of acupuncture with medication showed a moderate level of evidence due to the large effect size in the intervention groups compared to the control groups. In other subgroup analyses, group allocation was not properly designed, and hence, the level of evidence was low. The level of evidence for single subgroup analysis was low because it had a large effect size but a small sample size, which decreased the quality of assessment. In the analysis of the effective rate between combined acupuncture and medication and medication only groups, despite a large effect size, the level of evidence was low due to imprecision in the intervention groups.

**Table 2:** The level of evidence and Meta-Analysis of Outcomes

Variable	Overall effect				Studies (N)	Sample size (N)	Level of evidence
	MD or OR	95% CI	P	I^2^			
Acupuncture (sole) versus Medication							
VAS	−2.35	−2.84, −1.86	< 0.001	51%	5	305	Very low
Acupuncture (sole) versus Medication							
Effective rate	4.96	2.24, 10.99	< 0.001	0%	5	322	Low
Effective rate (used less than 10 points)	4.57	1.52, 13.72	0.007	0%	3	187	Low
Effective rate (used more than or equal10 points)	5.44	1.72, 17.19	0.004	0%	2	135	Moderate
Effective rate (used meridian points)	6.69	2.44, 18.31	< 0.001	0%	4	255	Moderate
Effective rate (not used meridian points)	3.03	0.83, 11.08	0.09	–	1	67	Low
Effective rate (less than a week)	5.79	1.16, 28.94	0.03	–	1	75	Moderate
Effective rate (between a week and two weeks)	3.85	1.18, 12.59	0.03	0%	2	127	Low
Effective rate (more than two weeks)	6.39	1.52, 26.87	0.01	0%	2	120	Moderate
Acupuncture (+ Medication) versus Medication							
Effective rate	6.68	1.11, 40.37	0.04	0%	2	130	Low

Abbreviations: *MD* Mean Difference, *OR* Odds Ratio, *CI* Confidence Interval, *VAS* Visual Analogue Scales

## Discussion

Occipital neuralgia refers to occipital pain originating in the suboccipital region and radiating to the occipital and temporal regions, and even as far as the frontal region. Among different types of headache, occipital neuralgia is the third most common after migraine and tension headache [29]. Although it is the major cause of headache in both adults and children, there has not been much research on occipital neuralgia, and this is thought to be related to difficulties ascertaining the exact incidence and prevalence, and understanding the precise pathology [30–32].

Occipital neuralgia is known to be caused by problems in the greater occipital nerves, lesser occipital nerves, or third occipital nerves, and examples of pathological causes include trauma, occipital nerve compression, and tumor diseases; however, the precise cause cannot be found in most cases [33]. Occipital neuralgia is not usually accompanied by structural abnormalities, and the occipital nerves are often sensitive due to irritation, and so the main purpose of treatment is to alleviate symptoms [8]. Specifically, if there is no improvement in symptoms after conservative treatment such as physical therapy, including thermotherapy, physiotherapy, yoga, and massage, or chiropractic treatment, the patient is given medication, such as opioid analgesics, non-opioid analgesics, anti-inflammatory agents, antispasmodics, or antidepressants [34]. However, some occipital neuralgia patients do not respond to conservative treatment alone, and so invasive methods are also used, such as Botox injection, occipital nerve block, or pulsed radiofrequency, and lately, surgical methods such as neuroablation or neurolysis [8]. Of these, occipital nerve block is simpler and safer than surgical treatment, and so it is considered the most appropriate treatment method [8].

In TOM, occipital neuralgia is treated using methods with few adverse effects, such as acupuncture, electroacupuncture, pharmacoacupuncture, moxibustion, and *chuna* manual therapy. Currently, TOM institutions treat large numbers of occipital neuralgia patients using acupuncture, moxibustion, electroacupuncture, and *chuna*, and patients who have received conventional medicine, such as medication, but experienced no improvement in symptoms often visit TOM institutions. There have already been many studies reporting that acupuncture stimulates secretion of opioid peptides such as β-endorphins, enkephalins, and dynorphins, and that these have an analgesic effect via their actions in the central nervous system [35, 36]. However, the mechanisms of acupuncture in occipital neuralgia remain unclear, and research investigating the effects of TOM, including acupuncture, on occipital neuralgia are still limited to RCTs. Hence, in this study, in order to improve the level of evidence for acupuncture, which can be considered an effective treatment modality with few adverse effects, we conducted a systematic literature review and meta-analysis of studies investigating the effects of acupuncture on occipital neuralgia.

We analyzed a total of 11 studies, classified by intervention; there were 9 studies using only acupuncture as the intervention, and 2 studies using combination therapy, including acupuncture, as the intervention.

The most commonly used acupuncture points were GB 20 and the *ashi* points, followed by EX-B2. GB 20 and EX-B2 are thought to have been used commonly because they are located in the occipital region, which coincides with the distribution of the occipital nerves. In particular, GB20 is located inferior to the occipital bone, in a depression between the insertion of sternocleidomastoid and trapezius; thus, it is directly related to the distribution of the nerves involved in occipital neuralgia, and this is the reason it is used in a lot of studies. EX-B2 is thought to have been used because its location coincides with the beginning parts of the greater and lesser occipital nerves. The *ashi* points are thought to have been used commonly in treating occipital neuralgia because these points refer to the areas where pain is experienced.

In the assessment of the risk of bias, the 11 RCTs included in this analysis were mostly rated as ‘unclear’ or ‘high’; this is thought to have been mostly because the nature of acupuncture research makes blinding difficult, and because acupuncture causes fewer adverse effects than other treatment modalities.

According to the meta-analysis of the 6 RCTs providing VAS results, combined acupuncture with medication was significantly more effective at alleviating pain than medication alone. However, the meta-analysis of acupuncture alone was limited because the heterogeneity of the studies was somewhat high. According to the meta-analysis of the 7 RCTs providing results for effective rate, treatment using acupuncture was significantly more effective at alleviating pain compared to medication alone, and the heterogeneity of the studies was also low.

We divided the acupuncture only studies into subgroups based on three criteria, and we did not detect any heterogeneity, which can be caused by subgrouping. Using ORs, when we analyzed the effective rate for subgroups classified by the number of acupuncture points, the ORs for the < 10 points and ≥ 10 points subgroups were similar (4.57 and 5.44, respectively). When we analyzed effective rate for subgroups classified by the type of acupuncture points, acupuncture based on the meridian theory showed a higher OR than acupuncture not related to the meridian theory (6.69 and 3.03, respectively). This indicates that the type of acupuncture points is more important than the number of acupuncture points when treating occipital neuralgia.

In terms of the duration of acupuncture treatment, according to the subgroup analysis (Additional file 5), > 2 weeks of treatment showed a higher OR than the other two durations. This suggests that acupuncture treatment for more than 2 weeks might be most effective in occipital neuralgia.

The fact that these analyses are based on studies with poor blinding or small numbers of participants has a negative impact on the level of evidence. Nevertheless, because the results comprehensively show that acupuncture had a stronger effect than medication, the level of evidence was found to be moderate in several analyses (Table 2). The level of evidence was also rates as low or very low for some analyses including studies with issues in the subject selection process.

Our study adhered to the systematic review and meta-analysis design so that it would provide a high level of evidence. Nevertheless, the study had several limitations. The 11 studies used in the final analysis were all conducted in China. Even though a large number of worldwide doctors are treating occipital neuralgia patients with acupuncture in clinical practice [37–40], in addition to traditional Korean medicine practitioners in Korea, little research is being conducted in this area, and so we were unable to analyze studies from countries other than China. Thus, in the future, research on acupuncture treatment for occipital neuralgia will need to be performed in more countries.

When we analyzed subgroups based on the acupuncture points used for acupuncture only intervention groups, homogeneity was low. However, in counting the number of acupuncture points, we treated the left and right sides as one, without distinction, which could be considered a limitation.

We could not find an explanation pertaining to the choosing of acupoints according to acupuncture theory in any of the studies. Although numerous acupoints have been used in studies related to occipital neuralgia care, which acupoints are to be used depends on the doctor’s knowledge, which could be considered a limitation.

Although dry needling, pharmacopuncture, and electroacupuncture are considered similar to acupuncture, studies using these techniques in the intervention group were excluded in our analyses. Since dry needling is not based on TOM or meridian theory, pharmacoacupuncture involves injecting drugs, and electroacupuncture involves artificial electrical stimulation, we treated these methods as being different from acupuncture therapy; this could be a potential limitation.

In fact, funnel plots used as a tool to judge the publication bias were difficult to apply in this study because of fewer studies. However, the publication bias has been judged as it has already been reflected in imprecision, inconsistency, large effect, etc. [41, 42]. Therefore, the publication bias was not evaluated in GRADE evaluation, and it was set to 0 points.

## Conclusion

This study has value as the first systematic literature review and meta-analysis providing evidence of the effects of acupuncture in treating occipital neuralgia. Although acupuncture only and combined acupuncture treatments showed significant effects compared to medication, the results of this study are inconclusive. We expect that our data could be used in acupuncture research and clinical practice for occipital neuralgia, and we believe that further, high-quality studies will be required in this field.

## Supplementary information

**Additional file 1.** Search strategy.

**Additional file 2.** Meta-analysis of Acupuncture with Medication vs. Medication (Effective rate).

**Additional file 3.** The subgroup Meta-analysis of Acupuncture vs. Medication according to the number of acupuncture points used (Effective rate).

**Additional file 4.** The subgroup Meta-analysis of Acupuncture vs. Medication according to the type of acupuncture points used (Effective rate).

**Additional file 5.** The subgroup Meta-analysis of Acupuncture vs. Medication according to the duration of treatment (Effective rate).

## Data Availability

The datasets supporting the conclusions of this article are included within the article.

## Abbreviations

AT: Acupuncture treatment
BRS-6: The 6-point Behavioral Rating Scale
CI: Confidence intervals
EA: Electrical acupuncture
MA: Manual acupuncture
MD: Mean difference
OR: Odds ratio
RCTs: Randomized controlled trials
RR: Relative risks
TCM: Traditional Oriental medicine
VAS: Visual analogue scale
