# Supplementary material for: The effects of acupuncture on occipital neuralgia: a systematic review and meta-analysis
Source: BMC Complement Med Ther. 2020 Jun 3;20:171. doi: 10.1186/s12906-020-02955-y (PMC7268636; doi:10.1186/s12906-020-02955-y)
Supplement: Supplementary file 1 — Additional file 1. Search strategy. [file 12906_2020_2955_MOESM1_ESM.docx]

Appendix 1 Search strategy

|  | | **Ovid EMBASE to 2019 December** | |
| --- | --- | --- | --- |
|  | | Searches | Results |
| **1** | exp neuralgia/ | | 102259 |
| **2** | exp secondary headache/ | | 1170 |
| **3** | cervicogenic headache.mp. | | 876 |
| **4** | occipital neuralgia.mp. | | 683 |
| **5** | exp headache/ | | 209251 |
| **6** | exp nerve compression/ | | 13251 |
| **7** | occipital nerve entrapment.mp. | | 8 |
| **8** | 2 or 3 or 4 or 6 or 7 | | 15582 |
| **9** | exp acupuncture/ | | 45596 |
| **10** | exp acupuncture needle/ | | 1164 |
| **11** | exp acupressure/ | | 2144 |
| **12** | 9 or 10 or 11 | | 45755 |
| **13** | 8 and 12 | | 154 |
| **14** | clinical trial/ | | 963202 |
| **15** | randomized controlled trial/ | | 584142 |
| **16** | randomization/ | | 85640 |
| **17** | single blind procedure/ | | 37464 |
| **18** | double blind procedure/ | | 168378 |
| **19** | crossover procedure/ | | 61707 |
| **20** | placebo/ | | 344359 |
| **21** | randomi?ed controlled trial$.tw. | | 217847 |
| **22** | rct.tw. | | 35319 |
| **23** | random allocation.tw. | | 2016 |
| **24** | randomly allocated.tw. | | 34130 |
| **25** | allocated randomly.tw. | | 2513 |
| **26** | (allocated adj2 random).tw. | | 889 |
| **27** | single blind$.tw. | | 24094 |
| **28** | double blind$.tw. | | 205095 |
| **29** | ((treble or triple) adj blind$).tw. | | 1094 |
| **30** | placebo$.tw. | | 301102 |
| **31** | prospective study/ | | 572700 |
| **32** | 14 or 15 or 16 or 17 or 18 or 19 or 20 or 21 or 22 or 23 or 24 or 25 or 26 or 27 or 28 or 29 or 30 or 31 | | 2147392 |
| **33** | 13 and 32 | | 36 |

|  | | **Ovid AMED to 2019 December** | |
| --- | --- | --- | --- |
|  | | Searches | Results |
| **1** | exp Neuralgia/ | | 252 |
| **2** | occipital neuralgia.mp. | | 12 |
| **3** | exp Headache/ | | 1430 |
| **4** | cervicogenic headache.mp. | | 68 |
| **5** | occipital nerve.mp. | | 9 |
| **6** | occipital headache.mp. | | 3 |
| **7** | occiput.mp. | | 59 |
| **8** | 1 or 2 or 3 or 4 or 5 or 6 or 7 | | 1740 |
| **9** | exp Acupuncture/ | | 3402 |
| **10** | exp Acupuncture therapy/ | | 8371 |
| **11** | Traditional medicine chinese/ | | 5985 |
| **12** | exp Acupressure/ | | 332 |
| **13** | 9 or 10 or 11 or 12 | | 14718 |
| **14** | 8 and 13 | | 353 |
| **15** | exp Clinical trials/ | | 4259 |
| **16** | 14 and 15 | | 20 |

|  | | **Cochrane Central Register of Controlled Trials : 2019 December** | |
| --- | --- | --- | --- |
|  | | Searches | Results |
| **1** | (occipital neuralgia):ti,ab,kw | | 43 |
| **2** | (occipital nerve entrapment syndrome):ti,ab,kw | | 1 |
| **3** | (cervicogenic headache):ti,ab,kw | | 201 |
| **4** | (occipital nerve):ti,ab,kw | | 334 |
| **5** | (occipital headache):ti,ab,kw | | 221 |
| **6** | MeSH descriptor: [Post-Traumatic Headache] explode all trees | | 81 |
| **7** | #1 or #2 or #3 or #4 or #9 or #10 | | 606 |
| **8** | (acupuncture):ti,ab,kw | | 13989 |
| **9** | (needle):ti,ab,kw | | 12620 |
| **10** | (needling):ti,ab,kw | | 2025 |
| **11** | (acupressure):ti,ab,kw | | 1233 |
| **12** | #5 or #6 or #7 or #8 | | 26439 |
| **13** | #7 and #12 | | 72 |

|  | CNKI | Date : December. 31. 2019 |
| --- | --- | --- |
|  | Searches | Results |
| #1 | (SU='後頭神經痛' OR SU='枕神經痛' OR SU='枕神经痛' OR SU='後頭神経痛' OR SU='后头神経痛') AND (SU='鍼' OR SU='針' OR SU='针' OR SU='針刺' OR SU='针刺') AND (SU='随机' OR SU='对照') | 22 |

|  | Pubmed | Date : December. 31. 2019 |
| --- | --- | --- |
|  | Searches | Results |
| #1 | [(acupuncture[Title/Abstract] OR needle[Title/Abstract] OR needling[Title/Abstract] OR acupressure[Title/Abstract]) AND (occipital neuralgia[Title/Abstract] OR occipital nerve entrapment syndrome[Title/Abstract] OR cervicogenic headache[Title/Abstract])] | 39 |

|  | J-Stage | Date : December. 31. 2019 |
| --- | --- | --- |
|  | Searches | Results |
| #1 | Full Text : acupuncture AND occipital neuralgia AND random | 3 |
| #2  #3 | Full Text : acupuncture AND occipital neuralgia AND random | 0  58 |
|  | Full Text : 針 AND 後頭神經痛 AND 臨床 |  |

|  | Acutrials | Date : December. 31. 2019 |
| --- | --- | --- |
|  | Searches | Results |
| #1 | Title contains occipital neuralgia AND Title contains acupuncture | 1 |
| #2  #3 | Title contains occipital neuralgia AND Title contains needle | 0  1 |
|  | Title contains occipital neuralgia AND Title contains needling |  |
| #4 | Title contains occipital neuralgia AND Title contains acupressure | 0 |
| #5 | Title contains cervicogenic headache AND Title contains acupuncture | 1 |
| #6 | Title contains cervicogenic headache AND Title contains needle | 1 |
| #7 | Title contains cervicogenic headache AND Title contains needling | 0 |
| #8 | Title contains cervicogenic headache AND Title contains acupressure | 0 |
| #9 | Title contains occipital nerve entrapment AND Title contains acupuncture | 0 |
| #10 | Title contains occipital nerve entrapment AND Title contains needle | 0 |
| #11 | Title contains occipital nerve entrapment AND Title contains needling | 0 |
| #12 | Title contains occipital nerve entrapment AND Title contains acupressure | 0 |
| #13 | Subject contains occipital neuralgia AND Subject contains acupuncture | 0 |
| #14 | Subject contains occipital neuralgia AND Subject contains needle | 0 |
| #15 | Subject contains occipital neuralgia AND Subject contains needling | 0 |
| #16 | Subject contains occipital neuralgia AND Subject contains acupressure | 0 |
| #17 | Subject contains cervicogenic headache AND Subject contains acupuncture | 0 |
| #18 | Subject contains cervicogenic headache AND Subject contains needle | 0 |
| #19 | Subject contains cervicogenic headache AND Subject contains needling | 1 |
| #20 | Subject contains cervicogenic headache AND Subject contains acupressure | 0 |
| #21 | Subject contains occipital nerve entrapment AND Subject contains acupuncture | 0 |
| #22 | Subject contains occipital nerve entrapment AND Subject contains needle | 0 |
| #23 | Subject contains occipital nerve entrapment AND Subject contains needling | 0 |
| #24 | Subject contains occipital nerve entrapment AND Subject contains acupressure | 0 |

|  | KISS | Date : December. 31. 2019 |
| --- | --- | --- |
|  | Searches | Results |
| #1 | 전체 = 침 AND 전체 = 후두신경통 | 0 |
| #2  #3 | 전체 = 침 AND 전체 = 경추성두통 | 0  0 |
|  | 전체 = occipital neuralgia AND 전체 = acupuncture |  |
| #4 | 전체 = occipital neuralgia AND 전체 = needle | 1 |
| #5 | 전체 = cervicogenic headache AND 전체 = acupuncture | 3 |
| #6 | 전체 = cervicogenic headache AND 전체 = needle | 0 |

|  | RISS | Date : December. 31. 2019 |
| --- | --- | --- |
|  | Searches | Results |
| #1 | 전체 : 후두신경통 <AND> 전체 : 침 | 0 |
| #2  #3 | 전체 : 경추성두통 <AND> 전체 : 침 | 0  0 |
|  | 전체 : acupuncture <AND> 전체 : occipital neuralgia |  |
| #4 | 전체 : acupuncture <AND> 전체 : cervicogenic headache | 5 |
| #5 | 전체 : needle <AND> 전체 : occipital neuralgia | 2 |
| #6 | 전체 : needle <AND> 전체 : cervicogenic headache | 0 |

|  | NDSL | Date : December. 31. 2019 |
| --- | --- | --- |
|  | Searches | Results |
| #1 | 전체〓후두신경통 \| 경추성두통 AND 전체〓침 | 1 |
| #2 | 전체〓occipital neuralgia \| cervicogenic headache AND 전체〓Acupuncture | 25 |

|  | OASIS | Date : December. 31. 2019 |
| --- | --- | --- |
|  | Searches | Results |
| #1 | 후두신경통 AND 침 | 0 |
| #2  #3 | 경추성두통 AND 침 | 0  0 |
|  | occipital neuralgia AND acupuncture |  |
| #4 | cervicogenic headache AND acupuncture | 0 |

|  | JAR | Date : December. 31. 2019 |
| --- | --- | --- |
|  | Searches | Results |
| #1 | 후두신경통 | 0 |
| #2  #3 | 경추성두통 | 0  0 |
|  | occipital neuralgia |  |
| #4 | cervicogenic headache | 1 |
