# Supplementary figures and images for: The effects of acupuncture on occipital neuralgia: a systematic review and meta-analysis
Source: BMC Complement Med Ther. 2020 Jun 3;20:171. doi: 10.1186/s12906-020-02955-y (PMC7268636; doi:10.1186/s12906-020-02955-y)

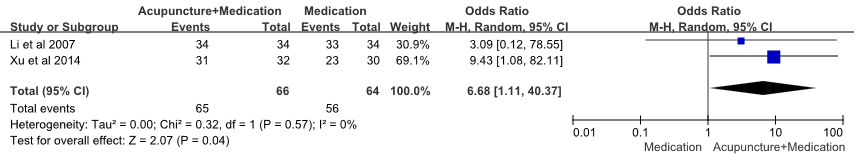

Supplement: Supplementary file 2 — Additional file 2. Meta-analysis of Acupuncture with Medication vs. Medication (Effective rate). [file 12906_2020_2955_MOESM2_ESM.tif]

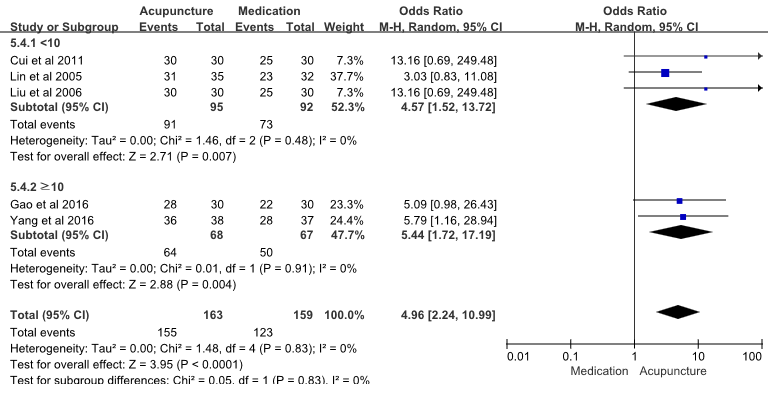

Supplement: Supplementary file 3 — Additional file 3. The subgroup Meta-analysis of Acupuncture vs. Medication according to the number of acupuncture points used (Effective rate). [file 12906_2020_2955_MOESM3_ESM.tif]

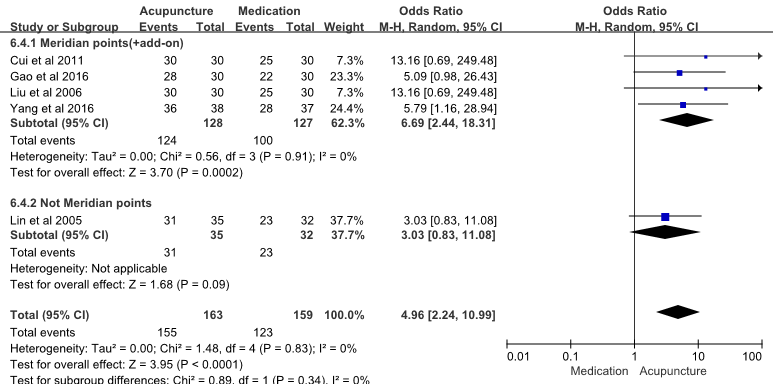

Supplement: Supplementary file 4 — Additional file 4. The subgroup Meta-analysis of Acupuncture vs. Medication according to the type of acupuncture points used (Effective rate). [file 12906_2020_2955_MOESM4_ESM.tif]

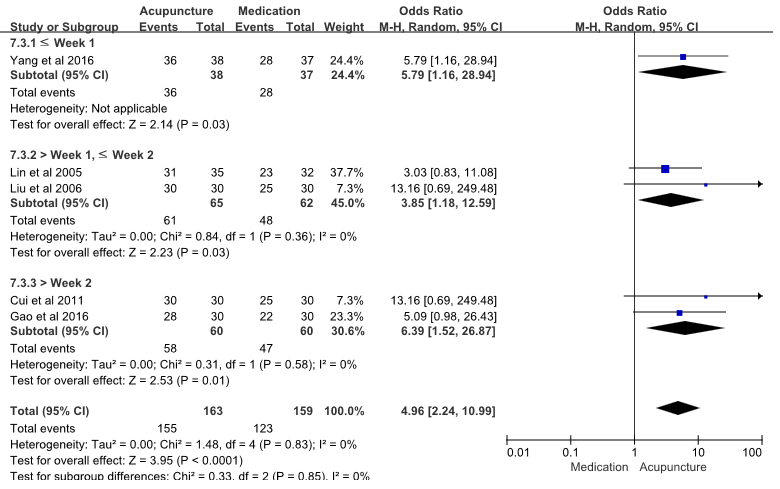

Supplement: Supplementary file 5 — Additional file 5. The subgroup Meta-analysis of Acupuncture vs. Medication according to the duration of treatment (Effective rate). [file 12906_2020_2955_MOESM5_ESM.tif]
